# Combining Endovascular Coiling and Open Evacuation for a Delayed-Onset Ruptured Post-traumatic Pseudoaneurysm of the Distal Paracentral Pericallosal Artery Branch

**DOI:** 10.7759/cureus.43880

**Published:** 2023-08-21

**Authors:** Manisha Koneru, Clint Badger, Alan R Turtz, Hamza Shaikh

**Affiliations:** 1 Neurointerventional Surgery, Cooper Medical School of Rowan University, Camden, USA; 2 Neurosurgery, Cooper University Health Care, Camden, USA; 3 Neurointerventional Surgery, Cooper University Health Care, Camden, USA

**Keywords:** traumatic pseudoaneurysm, intracranial hematoma, frontal craniotomy, endovascular coil embolization, blunt cervical trauma

## Abstract

Cerebrovascular pseudoaneurysm development and rupture is a rare, delayed sequelae of trauma. We present a case of a female patient in her sixties who presented after a fall without evidence of vascular injury on imaging. However, after one week, repeat imaging due to an abrupt change in mental status revealed a ruptured pseudoaneurysm, which was treated with a combination of coil embolization and open surgical evacuation of associated intracranial hematoma. This case illustrates the importance of continued surveillance beyond the acute traumatic period to identify late-onset complications in trauma patients requiring emergent treatment.

## Introduction

There is a high index of suspicion for cerebrovascular injury in cases of penetrating trauma, but it may be missed in patients presenting with blunt head and neck traumatic injuries, which are considered to be low mechanisms [1,2]. Vascular abnormalities, such as intracranial aneurysms, hematomas, carotid-cavernous fistulas, pseudoaneurysms, and vessel lacerations, can develop secondary to both penetrating and blunt head trauma [3,4]. Although computed tomography angiography (CTA) is the imaging modality of choice to detect post-traumatic cerebrovascular abnormalities, it is often not performed in cases of mild trauma [4,5].

Post-traumatic aneurysms and pseudoaneurysms may be treated with various endovascular methods to stabilize the compromised vessel and prevent subsequent rupture [4,6-8]. If undetected and untreated, subsequent rupture and hemorrhage can occur, presenting clinically as an acute change in neurological status [1,4,8]. Treatment includes surgical evacuation with microvascular repair [7,9]. Delayed rupture from undetected post-traumatic intracranial aneurysms and pseudoaneurysms is associated with a 30% to 50% mortality rate [3,6,10]. Consequently, extended surveillance and urgent intervention are critical for trauma patients at risk for precipitating late-onset cerebrovascular complications.

We presented a patient with a delayed-onset, post-traumatic development and rupture of a distal paracentral pericallosal artery branch pseudoaneurysm. The patient was treated using a combined endovascular and open-surgical approach, resulting in significant clinical recovery. This case was previously presented as a meeting poster at the Society of NeuroInterventional Surgery 20th Annual Meeting and Fellows Course from July 31, 2023, to August 4, 2023.

## Case presentation

A 64-year-old female with a history of insulin-dependent diabetes mellitus, hypertension, and hyperlipidemia on long-term aspirin antiplatelet therapy was admitted to the emergency department after falling down the stairs. She had a normal level of consciousness. She endorsed extensive acute pain. Laboratory values on admission were notable for elevated blood ethanol level that was five times the upper level of normal.

**Figure 1:**
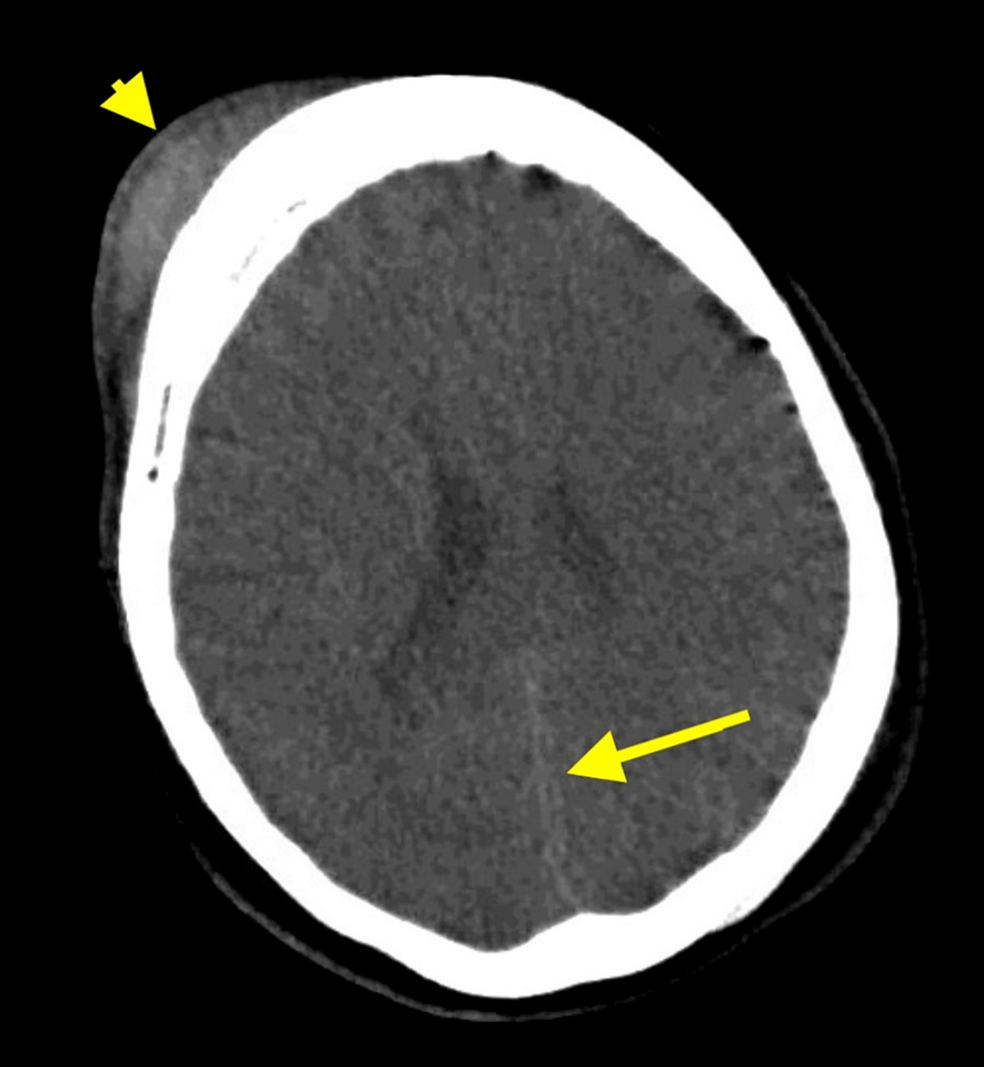
CT head without contrast on admission. An axial view of CT of the head without contrast demonstrates a large right frontal scalp hematoma (arrowhead) and 3 mm interhemispheric subdural hematoma (arrow) without midline shift. CT, computed tomography

Initial computed tomography (CT) without contrast of the head, facial bones, and cervical spine and CTA of the head and neck were performed. CT of the head demonstrated a large right frontal scalp hematoma, 3 mm interhemispheric subdural hematoma, and scattered areas of subarachnoid hemorrhage in both hemispheres (Figure 1). There was no evidence of midline shift, hydrocephalus, effacement of basal cisterns, or other vascular injury. No acute abnormalities were seen on the remainder of the imaging. 

She was also found to have an acute distal fracture of the right radius, which was managed in the interim with a closed reduction. One day after admission, she demonstrated no neurological abnormalities and behavior consistent with normal baseline function. Consequently, she was recommended to follow up in the outpatient setting with a repeat CT of the head in four weeks to monitor the stability of post-traumatic multicompartmental intracranial hemorrhages.

**Figure 2:**
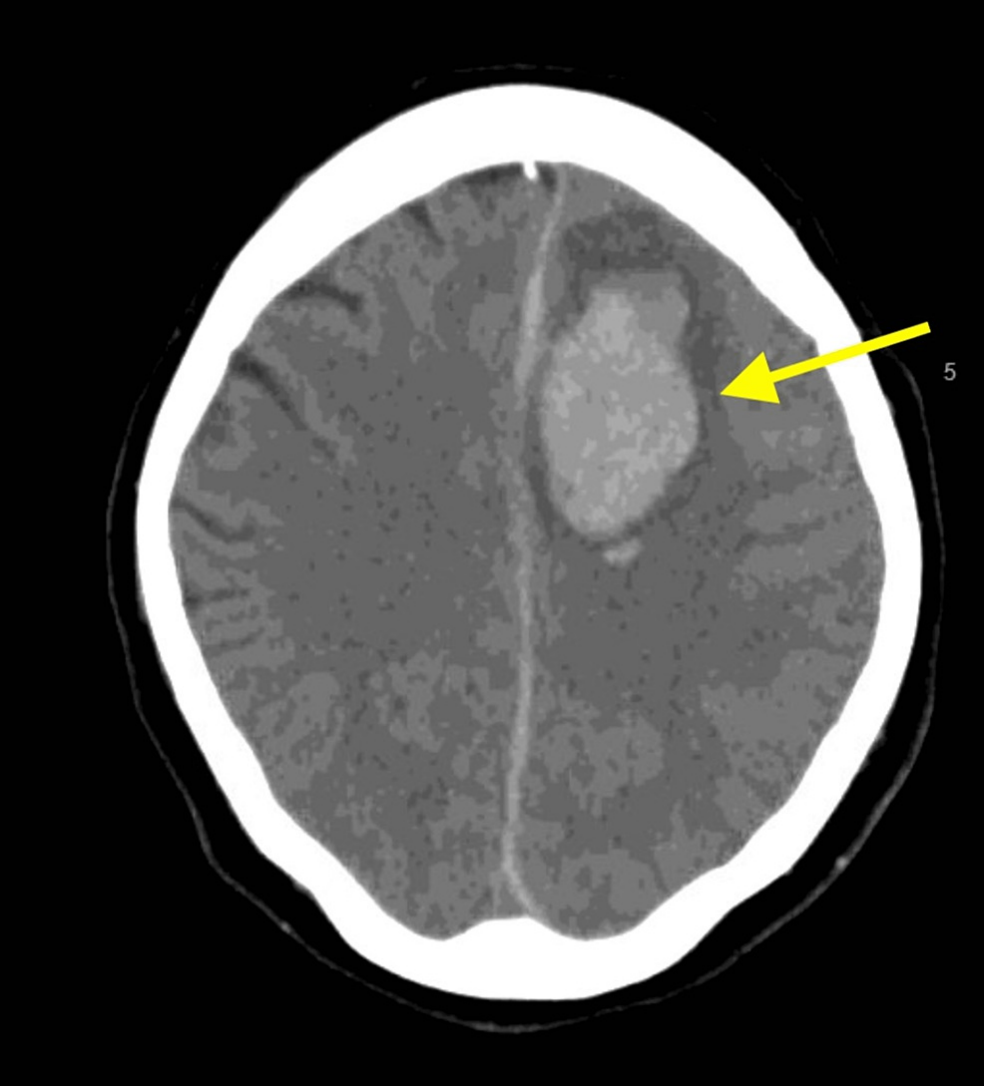
CT head without contrast after mental status change. An axial view of the CT of the head without contrast demonstrates a 4 cm left frontal parenchymal hematoma (arrow) with rightward subfalcine herniation. CT, computed tomography

**Figure 3:**
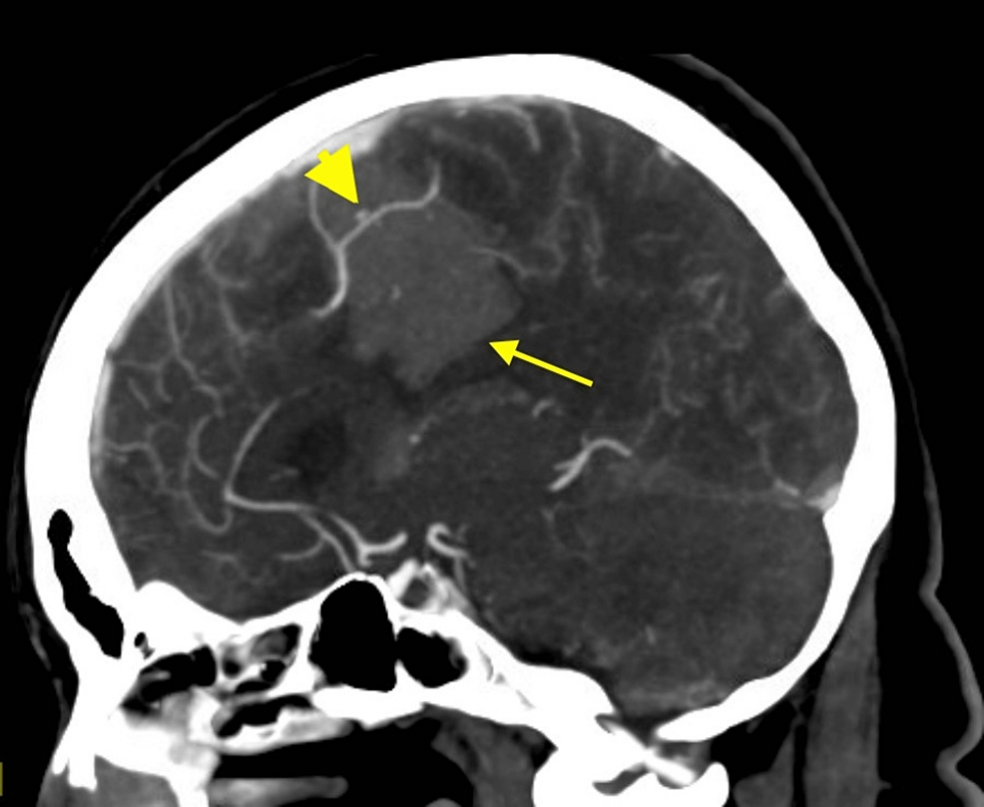
CTA of the head and neck showing ruptured pseudoaneurysm. A sagittal view of CTA of the head and neck on the arterial phase demonstrates a 2 mm ruptured pseudoaneurysm (arrowhead) within a left frontal parenchymal hematoma (arrow). CTA, computed tomography angiography

She underwent an open reduction and internal fixation of the right distal radius on the fourth day of admission. On the fifth day of admission, she endorsed pain localized to the right forehead. Laboratory results were notable for a decrease in hemoglobin from admission by approximately 1 g/dL. Within a few hours after endorsing the headache, she had sudden onset of decreasing level of consciousness with aphasia, prompting follow-up imaging. The CT of the head without contrast demonstrated a 4 cm left frontal parenchymal hematoma with rightward subfalcine herniation (Figure 2). The CTA of the head and neck demonstrated a vascular outpouching consistent with a 2 mm ruptured pseudoaneurysm within the left frontal parenchymal hematoma on the arterial phase (Figure 3). Signal enhancement within the hematoma on the venous phase was concerning for active extravasation. 

**Figure 4:**
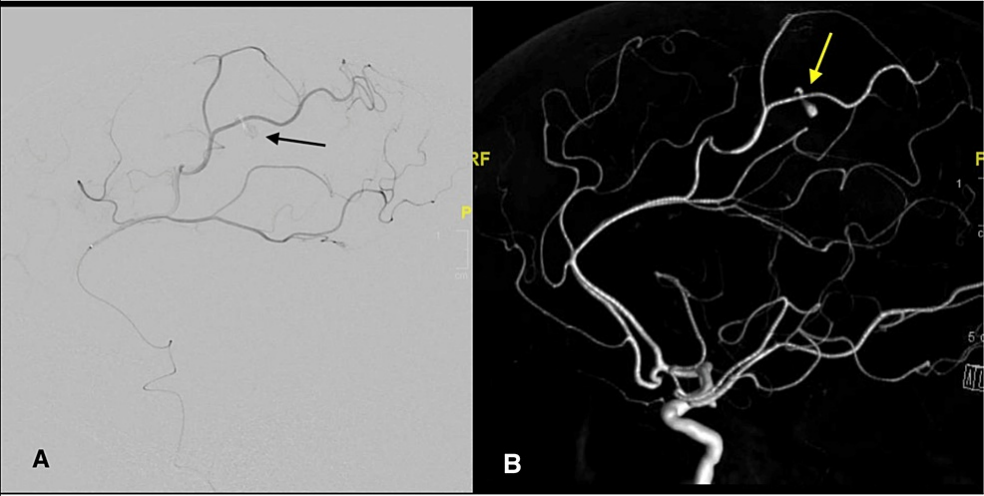
DSA and 3D tumble showing intracranial pseudoaneurysm. (A) DSA demonstrating a 3.2 mm × 2.0 mm × 1.8 mm pseudoaneurysm with active contrast extravasation (arrow) in the distal paracentral pericallosal branch of the left anterior cerebral artery. (B) 3D tumble reconstruction demonstrating pseudoaneurysm (arrow) in the distal paracentral pericallosal branch of the left anterior cerebral artery. 3D, three-dimensional; DSA, digital subtraction angiography

She was emergently taken to the neurointerventional suite for potential embolization to tamponade active bleeding. Using a 0.038-inch Glidewire guide wire (Terumo Medical Corporation, Somerset, NJ, USA), a 5-French Simmons two-guide catheter (Merit Medical Systems Inc., South Jordan, UT, USA) was used to selectively catheterize vessels for angiography. Cerebral angiography identified a 3.2 cm × 2.0 cm × 1.8 mm pseudoaneurysm with active contrast extravasation in the distal paracentral pericallosal branch of the left anterior cerebral artery (ACA) (Figures 4A-4B). 

**Figure 5:**
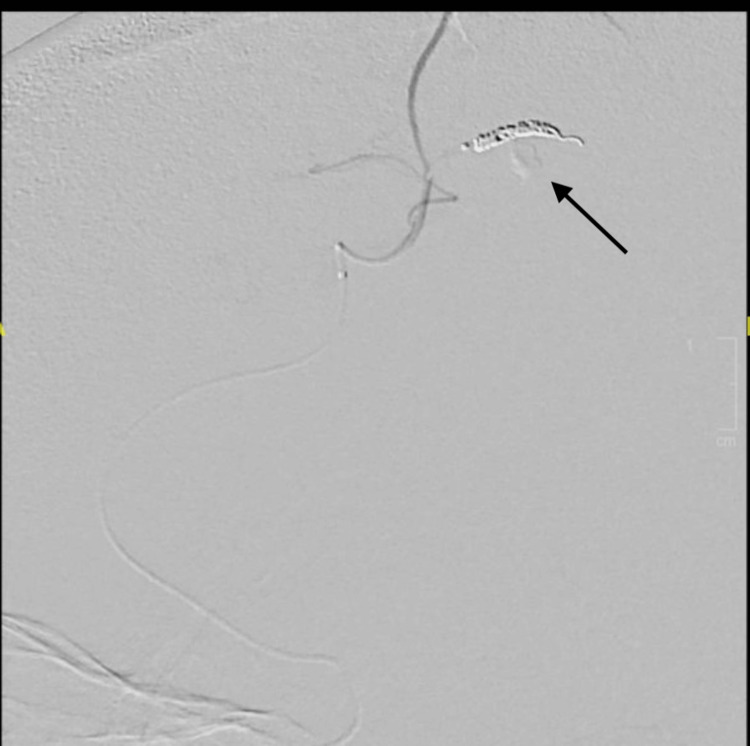
DSA after embolization. DSA demonstrating platinum coil embolization of pseudoaneurysm and distal parent artery (arrow). DSA, digital subtraction angiography

Following identification of the ruptured pseudoaneurysm, the guide catheter was exchanged for the Benchmark 071 access catheter (Penumbra, Place Alameda, CA, USA) to navigate to the left cervical internal carotid artery. The Aristotle 14 microwire (Scientia, West Valley City, UT, USA) and Excelsior SL-10 microcatheter (Stryker, Kalamazoo, MI, USA) construct was navigated to the left distal pericallosal branch of the ACA. Two sets of Target Helical Nano 1.5 mm × 4 cm platinum coils (Stryker) were deployed across the site of extravasation under continued fluoroscopic guidance. Angiography demonstrated embolization of the pseudoaneurysm and distal parent artery (Figure 5).

**Figure 6:**
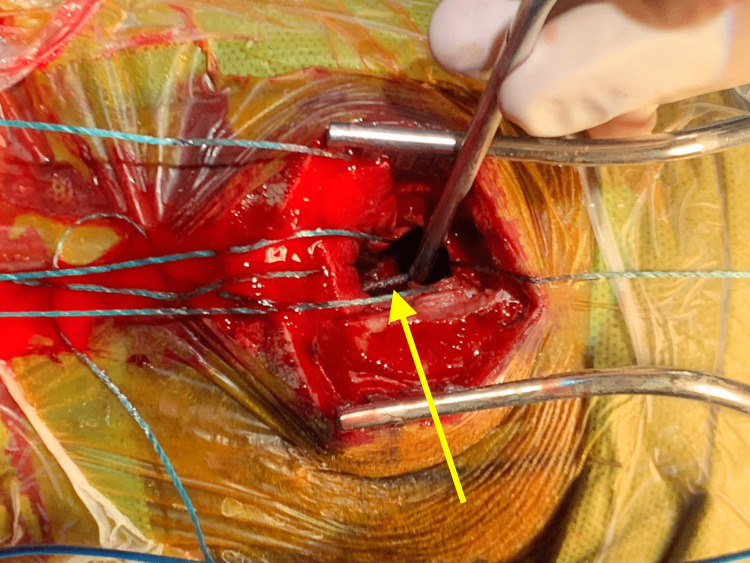
Endovascular coils visualized during open surgical hematoma evacuation. Left frontal craniotomy for evacuating associated clotted hematoma exposed endovascularly placed coils (arrow) in the left pericallosal artery.

She was then taken to the operating room for emergent evacuation of the hematoma. She underwent a left frontal craniotomy with evacuation of the clotted hematoma. During dissection and evacuation, endovascularly placed coils were visualized (Figure 6). 

Postoperatively, she gradually demonstrated improved neurological function. Follow-up cerebral angiogram one week after intervention demonstrated no residual pseudoaneurysm or extravasation. The hospital course was complicated by leukocytosis and fever due to a urinary tract infection. She was discharged to a subacute rehabilitation facility.

## Discussion

This case demonstrates that pseudoaneurysm development and rupture can be a delayed complication of trauma. Although initial screening CTA is performed for patients with nonpenetrating trauma meeting the Modified Denver Criteria, the initial CTA in our patient was negative [4,5]. Change in neurological status five days after the initial traumatic incident prompted repeat CTA, which revealed the ruptured pseudoaneurysm. Thus, there should be a strong consideration for delayed follow-up CTA in trauma patients at risk of precipitating late-onset complications.

Prior literature describes approaching post-traumatic pseudoaneurysms or aneurysms with either an endovascular coil embolization or surgical evacuation with microvascular repair, depending on whether there is rupture or extravasation [3,4,8-10]. Consideration for an approach to treatment includes the ability to endovascularly access the lesion and embolize it with maximal preservation of parental vessels [6]. Additionally, postoperative complication rates differ between the approaches. For example, a retrospective study identified a high incidence of infection, postoperative collections, infarcts, and vasospasm following open surgical repair of aneurysms [11]. On the other hand, a case series in the pediatric population described a significantly larger decrease in the rate of postintervention seizure at six months in patients with surgically treated intracranial distal arterial aneurysms than in the patients who were endovascularly treated [7].

Although several case reports describe individual successes with either endovascular or open surgical approaches in specific clinical contexts, there is still significant morbidity and mortality associated with treated post-traumatic aneurysms. Our case presents a hybrid approach combining endovascular and open surgical techniques that facilitated rapid control of the compromised vessel and relief from increased intracranial pressure due to the associated hematoma. Given the patient’s meaningful clinical recovery and lack of complications from the procedure, a hybrid endovascular and open approach may be a viable treatment option for post-traumatic aneurysms.

## Conclusions

Cerebrovascular pseudoaneurysms are a rare, delayed complication following blunt trauma. Continued close surveillance, including delayed follow-up CTA, is warranted for patients at risk for precipitating vascular pathology, especially if exhibiting signs of abnormal mental status or other decompensation in clinical status. An approach combining endovascular coiling and open surgical evacuation was successful to treat a ruptured pseudoaneurysm and hematoma in a distal anterior cerebral artery branch.
